# Increasing incidence and prevalence of metabolic syndrome in people living with HIV during the COVID-19 pandemic

**DOI:** 10.3389/fmed.2023.1220631

**Published:** 2023-09-18

**Authors:** Rebecka Papaioannu Borjesson, Laura Galli, Camilla Muccini, Andrea Poli, Tommaso Clemente, Martina Bottanelli, Nicola Gianotti, Silvia Nozza, Antonella Castagna, Vincenzo Spagnuolo

**Affiliations:** ^1^School of Medicine and Surgery, Vita-Salute San Raffaele University, Milan, Italy; ^2^Infectious Diseases Unit, San Raffaele Scientific Institute, Milan, Italy

**Keywords:** metabolic syndrome, HIV, diabetes, dyslipidemia, antiretroviral therapy, COVID-19

## Abstract

**Introduction:**

The aim of this study was to analyze the impact of COVID-19 pandemic restrictions on the prevalence and incidence of metabolic syndrome (MS), and to identify predictors of new MS cases in people living with HIV (PLWH).

**Methods:**

This cohort study included PLWH followed at the IRCCS San Raffaele, Milan, Italy, with at least one body mass index (BMI) determination during the pre-pandemic period (1 December 2018 to 29 February 2020) and the pandemic period (1 March 2020 to 31 May 2021). MS diagnosis was based on NCEP ATP III 2005 criteria. Univariable Poisson regression model was used to compare MS incidence rates. Univariable mixed linear models estimated the crude mean change in metabolic parameters during each time period. Multivariable Cox proportional hazards model was used to assess risk factors for MS.

**Results:**

This study included 1,564 PLWH, of whom 460 and 1,104 were with and without a diagnosis of MS, respectively, at the beginning of the pre-pandemic period, with an overall prevalence of MS of 29.4%. During the pre-pandemic period, 528/1,564 PLWH had MS, with a prevalence of 33.8% (95%CI = 31.5%–36.1%), while during the pandemic period, the number of PLWH with a diagnosis of MS increased to 628/1,564, with a prevalence of 40.2% (95%CI 37.8%–42.6%; McNemar’s test: *p* < 0.0001). Similarly, the MS incidence rate increased from 13.7/100 person-years of follow-up (PYFU; 95%CI = 11.7–16.0) in the pre-pandemic period to 18.5/100 PYFU (95%CI = 16.2–21.1) in the pandemic period (*p* = 0.004), with 201 subjects developing MS during the pandemic period. In addition, we observed a significant increase in the crude mean change during the pandemic period compared with the pre-pandemic period for: total cholesterol, LDL cholesterol, plasma glucose, blood pressure, and atherosclerotic cardiovascular disease (ASCVD) risk score. Finally, after adjustment for HIV risk factors, HBV, HCV, ART duration, duration of virologic suppression and use of INSTIs, age [adjusted hazard ratio (AHR) per 3 years older = 1.12 (95%CI = 1.08–1.17)], sex [AHR female vs. male = 0.62 (95%CI = 0.44–0.87)] and CD4+ cell count [AHR per 100 cells/μL higher = 1.05 (95%CI = 1.01–1.09)] were associated with the risk of MS.

**Conclusion:**

The COVID-19 pandemic affected the metabolic profile of PLWH and increased the prevalence and incidence of MS.

## Introduction

Metabolic syndrome (MS) is the coexistence in the same individual of metabolic risk factors for both type 2 diabetes and cardiovascular disease (CVD), such as abdominal obesity, insulin resistance, hypertension, and hyperlipidemia (1). MS is characterized by elevated levels of pro-inflammatory mediators (cytokines) leading to a pro-thrombotic and pro-inflammatory state that is commonly associated with accelerated atherosclerotic cardiovascular disease, hyperuricemia/gout, chronic kidney disease, and obstructive sleep apnea (2).

The global prevalence of MS varies according to geographic and sociodemographic factors (2), and this syndrome has been reported to be highly age-dependent (3).

In the United States, 35% of the general population and 50% of the population over 60 years of age have been diagnosed with MS according to the NCEP ATP III 2005 definition (4), with a higher prevalence in men (2).

In Europe, the difference between men and women persists, with a prevalence of MS of 41% and 38%, respectively (2).

Several studies have shown that the prevalence of MS in people living with HIV (PLWH) is similar to that in the general population (5–7), but metabolic disorders such as diabetes mellitus and dyslipidemia are more common in older PLWH (8, 9). As the life expectancy of PLWH is now almost identical to that of the general population and the average age of individuals with HIV is increasing (10), the prevalence of MS in this population is expected to continue to rise in the coming years.

The actual prevalence of MS in PLWH varies according to the different definitions and appears to be between 20 and 40% (11–13), and older age, male sex and a high CD4 cell count have been found to be associated with MS (13).

The prevalence of MS in the general population is increasing each year due to the increasing prevalence of sedentary lifestyles (1). Some studies have suggested how COVID-19 pandemic, due to confinement and restricted mobility, negatively affected the metabolic profile in the general population (14, 15).

To date, no studies have analyzed the prevalence and incidence of MS in PLWH during the COVID-19 pandemic.

Our aim was to analyze the impact of COVID-19 pandemic restrictions on the prevalence and incidence of MS and to identify its predictors in PLWH.

## Methods

This cohort study included PLWH regularly followed at the Infectious Diseases Unit of the San Raffaele Scientific Institute, Milan, Italy.

We considered in the analyses PLWH followed within two different time periods: pre-pandemic, from 1 December 2018 to 29 February 2020 and pandemic, between 1 March 2020 and 31 May 2021.

Only PLWH with at least one body mass index (BMI) measurement in each of these two time periods were included in the current study. PLWH with a previous diagnosis of MS at the beginning of the pre-pandemic period were excluded from the analyses but were included in the determination of MS prevalence in the different study periods.

The values at the beginning of each period for BMI and laboratory parameters were defined as the mean of the first two measurements collected at the start of the pre-pandemic or pandemic period, while during follow-up each additional measurement was considered; we used mean values, calculated on multiple baseline measurements, in order to get a more realistic picture of people’s health status at the start of each time period and avoid the phenomenon of regression to the mean.

According to BMI, PLWH included in the study were divided into three groups: normal weight (BMI < 25 Kg/m^2^), overweight (BMI ≥ 25 and <30 Kg/m^2^), and obese (BMI ≥ 30 Kg/m^2^).

Dyslipidemia was defined as the presence of at least one of the following: total cholesterol >190 mg/dL, LDL > 130 mg/dL, HDL < 40 mg/dL in men, < 50 mg/dL in women, triglycerides > 150 mg/dL, specific hypolipidemic treatment (fenofibrate and/or statins/ezetimibe).

The diagnosis of MS was based on the NCEP ATP III 2005 definition as the presence of at least three of the following criteria BMI ≥ 30 kg/m^2^ (as a surrogate for abdominal obesity); triglycerides (TG) ≥ 150 mg/dL (or use of fenofibrate); HDL cholesterol < 40 mg/dL in men, < 50 mg/dL in women (or use of statins or ezetimibe); fasting plasma glucose ≥ 100 mg/dL (or previous diagnosis of diabetes) (4). In contrast to the original NCEP ATP III 2005 definition, we have included statins, ezetimibe, and fibrates as specific lipid-lowering drugs, given the widespread use of these treatments in PLWH with metabolic abnormalities.

We decided to use BMI ≥ 30 kg/m^2^ as a surrogate marker of central obesity instead of waist circumference (15), as the latter was not available for the entire PLWH cohort.

### Statistical analysis

Results were described as median (interquartile range, IQR) or frequency (%). At univariate analysis, comparisons of individuals’ characteristics between antiretroviral treatment groups were performed using the chi-square/Fisher exact test for categorical variables and by use of the Wilcoxon rank sum test for continuous variables.

Prevalence was calculated at the end of each time period, as the proportion of people with MS at the end of each time period; prevalence proportions of the two time periods were compared using the McNemar’s test.

Univariable Poisson regression model was used to estimate and compare crude incidence rates (IR) of MS, calculated as the number of people who developed MS within each time period, divided by the total person-years of follow-up (PYFU) in that time period. In this analysis, follow-up was calculated from the date of the first visit of each time period until the date of MS or the end date of each period if they did not experience MS.

Univariable mixed linear models (MLM) with random slope and intercept for each patient were fitted to estimate crude mean changes in the considered laboratory parameters during each time period; slopes were reported with the corresponding 95% confidence intervals. For these analyses, follow-up started on the date of the first parameter determination and ended on the date of the last parameter determination of each time period and changes were calculated since the first available value of the considered time period.

Multivariable Cox regression model was used to assess factors associated with the risk of MS; adjusted hazard ratio (aHR) with the corresponding 95% confidence intervals (95%CI) were reported. The following time-fixed covariates, measured at the start of the pandemic period, known to have a potential effect on this outcome or those with *p* < 0.1 at univariable analysis were considered in the final multivariable model: age, sex, HIV risk factor, HBV co-infection, HCV co-infection, ART duration, duration of virological suppression, use of integrase strand inhibitors (INSTI) and CD4 T cells count. In multivariable analysis, follow-up started at the date of the first visit of the pre-pandemic period until the date of MS or the date of the last visit of the pandemic period if they did not experience MS.

All analyses were conducted using SAS statistical software version 9.4 (Statistical Analyses System Inc., Cary, NC, United States).

## Results

Of the 1,564 PLWH who met the inclusion criteria, 460 and 1,104 had been diagnosed with and without MS at the beginning of the pre-pandemic period, respectively.

**Figure 1:**
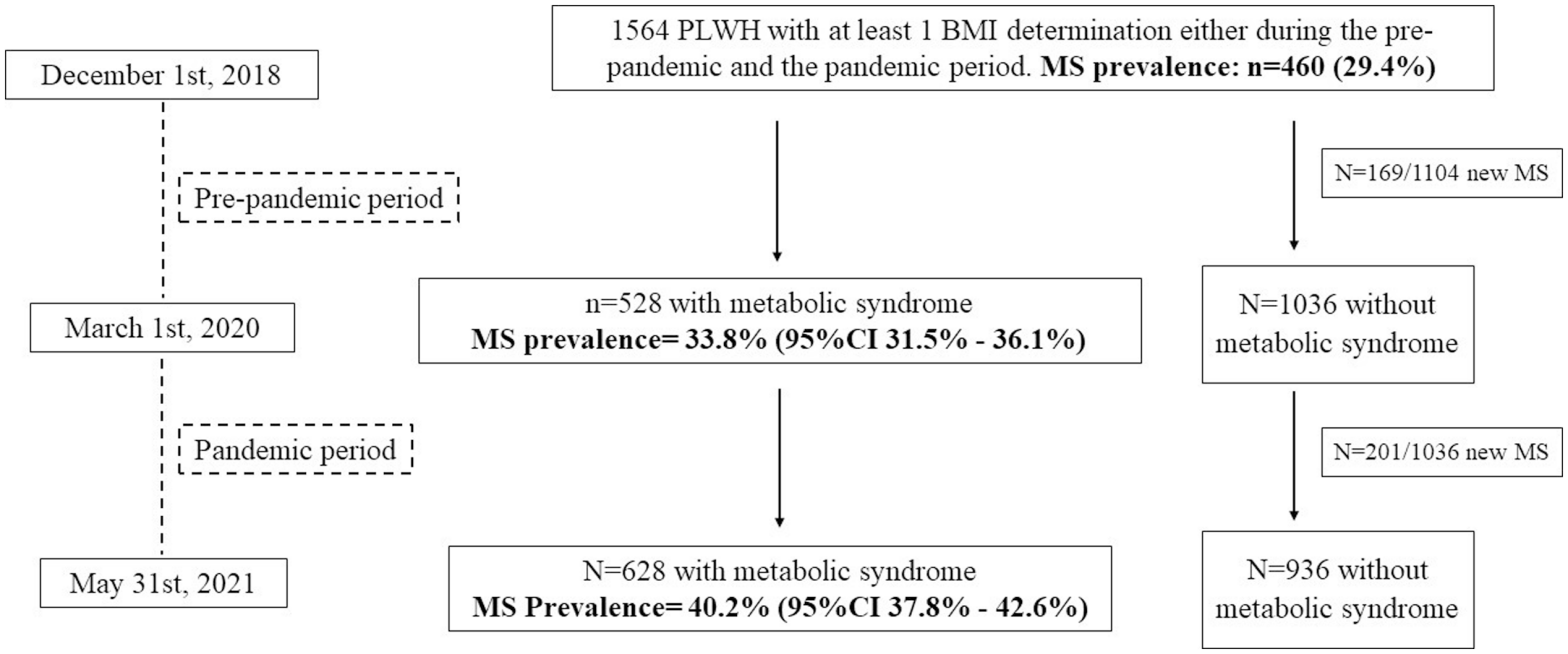
Distribution of PLWH included in the study and prevalence of metabolic syndrome (MS) during the two study periods.

The distribution of individuals and the prevalence of MS in the different study periods is shown in Figure 1.

At the beginning of the pre-pandemic period, the median age of the 1,104 individuals without a previous diagnosis of MS was 51.2 years (IQR = 43.5–55.7), 77% were male, and 45% were smokers. One thousand eight individuals (91.9%) had an undetectable viral load (defined as HIV RNA < 50 copies/mL), with a nadir CD4+ of 274 cells/uL (144–398) and a CD4+ cell count of 729 cells/uL (552–945).

A detailed description of the clinical characteristics, metabolic profile, cardiovascular risk factors and antiretroviral therapies at the beginning of the pre-pandemic and pandemic periods according to the development of MS is presented in Tables 1, 2, respectively.

**Table 1:** Clinical characteristics, metabolic profile, cardiovascular risk factors, and antiretroviral therapies at the beginning of the pre-pandemic period, according to the occurrence of metabolic syndrome (MS); only PLWH without a previous diagnosis of MS at the beginning of the pre-pandemic period were considered.

Variable	Overall (*n* = 1,104)	MS occurrence during the pre-pandemic period (*n* = 169)	Without MS occurrence during the pre-pandemic period (*n* = 935)	*p*-value^§^
Age, years	51.2 (43.3–55.7)	54.0 (49.3–58.6)	50.5 (42.4–55.0)	<0.0001
Male sex	845 (76.5%)	140 (82.8%)	705 (75.4%)	0.038
HIV risk factor				0.729
Heterosexual	198 (17.9%)	31 (18.3%)	167 (17.9%)	
MSM	530 (48%)	81 (47.9%)	449 (48%)	
Other	204 (18.5%)	27 (16%)	177 (18.9%)	
PWID	172 (15.6%)	30 (17.8%)	142 (15.2%)	
Years of HIV infection	16.1 (8.5–26.2)	21.6 (12.3–28.9)	15.4 (8.0–25.6)	<0.0001
Years of ARV	12.3 (5.6–20.9)	18.0 (9.6–22.8)	11.5 (5.4–20.6)	<0.0001
Nadir CD4+, cells/uL	274 (144–398)	246 (113–381)	278 (148–399)	0.097
CD4+, cells/uL	729 (552–945)	731 (549–951)	729 (556–937)	0.765
CD4/CD8	0.9 (0.6–1.3)	0.9 (0.6–1.3)	0.9 (0.7–1.3)	0.46
HIV-RNA < 50 copies/mL	1,008 (91.9%)	157 (92.9%)	851 (91.7%)	0.759
Duration of virological suppression, months	4.2 (1.7–8.9)	4.9 (1.8–10.2)	4.1 (1.7–8.7)	0.302
Positive HBsAg	67 (6.1%)	15 (8.9%)	52 (5.6%)	0.221
Positive Ab anti HCV	311 (28.2%)	49 (29.0%)	262 (28.0%)	0.467
Smokers	460 (45.4%)	65 (40.4%)	395 (46.3%)	0.165
Body mass index (BMI), Kg/m^2^	23.8 (21.6–25.9)	25.3 (22.8–28.1)	23.6 (21.5–25.5)	<0.0001
BMI ≥ 30Kg/m^2^	40 (3.6%)	26 (15.4%)	14 (1.5%)	<0.0001
Diabetes mellitus	14 (1.3%)	6 (3.6%)	8 (0.9%)	0.012
Systolic pressure, mmHg	124 (116–131.67)	130 (124–137)	123 (115–130)	<0.0001
Diastolic pressure, mmHg	79.5 (73–85.33)	85 (77.4–89.5)	78.5 (72.5–84.5)	<0.0001
Use of lipid-lowering drug	267 (24.2%)	69 (40.8%)	198 (21.2%)	<0.0001
HDL-cholesterol, mg/dL	50.33 (41.5–60.5)	46.5 (41.33–55.33)	51 (41.5–61.66)	0.002
LDL-cholesterol, mg/dL	121.5 (100–143)	130.5 (103.66–152)	120.5 (99.5–142)	0.015
Total cholesterol, mg/dL	182 (159–203.75)	187.5 (163.5–215)	181 (158.5–201)	0.005
Glucose, mg/dL	91.5 (85.7–97.3)	102.3 (95.0–106.5)	90.7 (85.3–95.0)	<0.0001
Triglycerides, mg/dL	102 (77–137)	150.5 (107–187)	97.7 (74.2–127)	<0.0001
ASCVD risk score	4.6 (1.9–8.8)	7.9 (4.4–12.6)	4 (1.5–8.1)	<0.0001
Current use of INSTIs	599 (54.3%)	91 (53.8%)	508 (54.3%)	0.933
Current use of PIs	257 (23.3%)	42 (24.9%)	215 (23.0%)	0.621
Current use of NNRTIs	282 (25.5%)	55 (32.5%)	227 (24.3%)	0.027

Results described by median (IQR) or frequency (%). ^§^Wilcoxon rank-sum test for continuous variable; Chi-square or Fisher exact test for categorical variables, as appropriate. HIV, human immunodeficiency virus; MSM, men who have sex with men; PWID, people who inject drugs; ARV, antiretroviral therapy; IDU, Intravenous drug users; CD4+, cluster of differentiation 4; HBsAg, Hepatitis B surface antigen; HCV, hepatitis C virus; HDL, high-density lipoprotein; LDL, low-density lipoprotein; ASCVD, atherosclerotic cardiovascular disease; INSTIs, integrase strand transfer inhibitors; PIs, protease inhibitors; NNRTIs, non-nucleoside reverse transcriptase inhibitors.

**Table 2:** Clinical characteristics, metabolic profile, cardiovascular risk factors, and antiretroviral therapies at the beginning of the pandemic period, according to the occurrence of metabolic syndrome (MS).

Variable	Overall (*n* = 1,036)	MS occurrence during the pandemic period (*n* = 201)	Without MS occurrence during the pandemic period (*n* = 835)	*p*-value^§^
CD4+, cells/uL	750 (567–946)	762 (567–1,042)	744 (568–933)	0.144
CD4/CD8	0.96 (0.66–1.27)	0.91 (0.6–1.29)	0.97 (0.69–1.27)	0.439
HIV-RNA < 50 copies/mL	949 (92.7%)	189 (94%)	760 (92.3%)	0.454
Body mass index (BMI), Kg/m^2^	24.1 (22.1–26.2)	25.3 (23.4–27.8)	23.8 (21.7–25.9)	<0.0001
BMI > 30Kg/m^2^	19 (1.8%)	9 (4.5%)	10 (1.2%)	<0.0001
Diabetes mellitus	14 (1.4%)	7 (3.5%)	7 (0.8%)	0.009
Systolic pressure, mmHg	126 (118–136)	133 (125–140)	124 (116–134)	<0.0001
Diastolic pressure, mmHg	81 (75–88)	86 (80.5–91.5)	80 (73.58–86)	<0.0001
Use of lipid-lowering drug	272 (26.3%)	75 (37.3%)	197 (23.6%)	<0.0001
HDL-cholesterol, mg/dL	50 (42–60.8)	47 (41–56)	51 (42.5–62.5)	0.0001
LDL-cholesterol, mg/dL	119.5 (99–141)	124 (106–144)	118 (97.5–140)	0.012
Total cholesterol, mg/dL	182.5 (162.6–206)	193 (172.3–214)	180 (161–203.5)	<0.0001
Glucose, mg/dL	94 (88.5–99)	102.5 (98.3–107.5)	92.5 (87.5–96.7)	<0.0001
Triglycerides, mg/dL	101 (77–137)	150 (93.5–181)	96.4 (75–126)	<0.0001
ASCVD risk score	4.3 (1.6–8.7)	7.0 (3.8–12.3)	3.7 (1.5–7.4)	<0.0001
Current use of INSTIs	660 (59.8%)	157 (60.4%)	503 (59.6%)	0.829
Current use of PIs	218 (19.7%)	54 (20.8%)	164 (19.4%)	0.656
Current use of NNRTIs	277 (25.1%)	75 (28.8%)	202 (23.9%)	0.120

Results described by median (IQR) or frequency (%). ^§^Wilcoxon rank-sum test for continuous variable; Chi-square or Fisher exact test for categorical variables, as appropriate. HIV, human immunodeficiency virus; HDL, high-density lipoprotein; LDL, low-density lipoprotein; ASCVD, atherosclerotic cardiovascular disease; INSTIs, integrase strand transfer inhibitors; PIs, protease inhibitors; NNRTIs, non-nucleoside reverse transcriptase inhibitors.

Regarding antiretroviral therapy, at the beginning of the pre-pandemic period, 523 individuals (47.4%) were on an INSTI-based regimen, 201 (18.2%) were on a PI-based regimen, 246 (22.3%) were on an NNRTI-based regimen and 134 (12.1%) on other ART regimens. In contrast, at the beginning of the pandemic period, 581 (52.6%), 166 (15.0%), and 236 (21.4%) subjects were taking an INSTI, a PI an NNRTI regimen and 121 (11.0%) on other ART regimens, respectively (McNemar’s test: *p* < 0.0001).

During the pre-pandemic period (median follow-up = 1.25 (1.25–1.25) years), 528/1,564 PLWH were diagnosed with MS, with a prevalence of 33.8% (95%CI = 31.5–36.1%), whereas during the pandemic period (median follow-up = 1.04 (1.03–1.06) years), 628/1,564 individuals were diagnosed with MS, with a prevalence of 40.2% (95%CI = 37.8–42.6%), *p* < 0.0001.

During the pre-pandemic period, 169 individuals developed MS, for a crude MS incidence rate of 13.7/100-PYFU (95%CI = 11.7–16.0); during the pandemic period, 201 individuals were newly diagnosed with MS, and the crude incidence rate increased to 18.5/100-PYFU (95%CI = 16.2–21.1; pre-pandemic vs. pandemic IR: *p* = 0.004).

Similarly, the prevalence and incidence of diabetes mellitus (DM) changed between the two periods: 10 individuals developed DM during the pre-pandemic period, for a crude DM incidence rate of 0.6/100-PYFU (95%CI = 0.3–1.1); during the pandemic period, 19 individuals were newly diagnosed with DM, and the crude incidence rate increased to 1.2/100-PYFU (95%CI = 0.8–1.9; pre-pandemic vs. pandemic IR: *p* = 0.063).

The prevalence of DM was 1.3% (95%CI = 0.7%–2.1%) in the pre-pandemic period and 1.4% (95%CI = 0.8%–2.3%) in the pandemic period.

During the pre-pandemic period, 168 subjects experienced an increase of BMI; 43 developed obesity (one of them was normal weight, while 42 subjects were already overweight), 125 subject who were normal weight before the pre-pandemic period became overweight during the pre-pandemic period.

The prevalence and incidence of overweight or obesity changed between the two periods: 138 individuals became overweight (n = 106) or obese (*n* = 32) during the pre-pandemic period, for a crude obesity incidence rate of 1.94/100-PYFU (95%CI = 1.37–2.74); during the pandemic period, 168 individuals became overweight (*n* = 125) or obese (*n* = 43), and the crude incidence rate of obesity increased to 2.84/100-PYFU (95%CI = 2.11–3.81; pre-pandemic vs. pandemic IR: *p* = 0.098).

Thus, during the pandemic period, 174 subjects were obese, 565 were overweight, and 825 PLWH were normal weight. The prevalence of obesity was 13.2% (95%CI = 11.4%–15.4%) in the pre-pandemic period and 15.8% (95%CI = 13.7%–18.0%) in the pandemic period; the prevalence of overweight/obesity was 61.8% (95%CI = 58.9%–64.6%) in the pre-pandemic period and 66.9% (95%CI = 64.1%–69.7%) in the pandemic period.

In addition, most of the metabolic parameters considered in our study showed a statistically significant crude mean change during both the pre-pandemic and pandemic periods (Table 3). The mean changes in these metabolic parameters during the pandemic period were generally greater than those estimated during the pre-pandemic period, particularly for total, HDL, and LDL cholesterol, plasma glucose, systolic and diastolic blood pressure, and atherosclerotic cardiovascular disease (ASCVD) risk score, as shown in Table 3.

**Table 3:** Univariable mixed linear models: crude mean changes in laboratory parameters during the pre-pandemic and the pandemic period.

Parameters	Crude mean value during pre-pandemia (95% CI)	Crude mean value during pandemia (95% CI)	Crude mean change (slope) per month during pre-pandemia (95% CI)	Crude mean change (slope) per month during pandemia (95% CI)	*p*-value (first vs. second period)
Weight, kg	71 (71–72)	72 (71–73)	+0.050 (0.036/ 0.064) *p* < 0.0001	+0.060 (0.046/ 0.073) *p* < 0.0001	0.614
Body mass index, kg/m^2^	23.8 (23.6–24.0)	24.1 (23.9–24.3)	+0.018 (0.013/ 0.023) *p* < 0.0001	+0.019 (0.015/ 0.024) *p* < 0.0001	0.730
Triglycerides, mg/dL	115 (112–119)	117 (114–121)	−0.047 (−0.235/0.141) *p* = 0.623	−0.102 (−0.287/ 0.082) *p* = 0.278	0.967
Total cholesterol, mg/dL	182 (180–184)	185 (183–187)	−0.190 (−0.292/ -0.087) *p* = 0.0003	+0.303 (0.202/ 0.405) *p* < 0.0001	<0.0001
HDL-cholesterol, mg/dL	52 (51–53)	53 (52–53)	−0.012 (−0.041/0.017) *p* = 0.414	+0.023 (−0.005/ 0.051) *p* = 0.001	0.003
LDL-cholesterol, mg/dL	122 (120–124)	119 (118–121)	−0.197 (−0.291/ -0.104) *p* < 0.0001	+0.019 (−0.073/0.110) *p* = 0.687	<0.0001
Glucose, mg/dL	92 (92–93)	95 (95–96)	−0.083 (−0.131/ -0.035) *p* = 0.0006	+0.217 (0.170/0.263) *p* < 0.0001	<0.0001
Systolic pressure, mmHg	125 (124–125)	126 (125–126)	+0.005 (−0.040/ 0.050) *p* = 0.831	+0.125 (0.082/ 0.169) *p* < 0.0001	0.0002
Diastolic pressure, mmHg	79 (79–80)	80 (80–81)	+0.009 (−0.027/ 0.045) *p* = 0.612	+0.100 (0.067/ 0.134) *p* < 0.0001	0.0003
ASCVD risk score	7.7 (7.2–8.3)	9.0 (8.4–9.6)	+0.020 (0.004/ 0.036) *p* = 0.016	+0.051 (0.035/ 0.067) *p* < 0.0001	0.005

CI, confidence interval; HDL, high-density lipoprotein; LDL, low-density lipoprotein; ASCVD, atherosclerotic cardiovascular disease.

Finally, in multivariable analysis, after adjusting for HIV risk factor, HBV co-infection, HCV co-infection, ART duration, duration of virological suppression and use of integrase strand inhibitors (INSTI), we observed that age [adjusted hazard ratio (aHR) per 3 years older = 1.13 (95%CI = 1. 08–1.18), *p* = 0.0007], sex [aHR; female vs. male = 0.69 (95%CI = 0.49–0.97), *p* = 0.032], BMI [overweight vs. normal weight: aHR = 1.546 (95%CI = 1.219–1.962), *p* = 0.0003]; obese vs. normal weight: [aHR = 7.468 (95%CI = 4.967–11.227), *p* < 0.0001], and CD4 cell count [aHR per 100 cells/μL increase = 1.05 (95%CI = 1.01–1.09), *p* = 0.008] were associated with the risk of MS.

## Discussion

In our study, both the prevalence and incidence of MS increased between the pre-pandemic period and the pandemic period. With the spread of the COVID-19 pandemic, lockdown conditions were introduced in many countries worldwide, particularly in Italy from March to June 2020. Even after these months of severe restrictions, the temporary closure of various public places, such as gyms and restaurants, forced changes in usual diet and physical activity (14).

Previous studies have shown how the restrictive measures during the COVID-19 outbreak affected both elderly and young healthy individuals (14–17), with a greater impact on those already suffering from metabolic disorders (14).

The increase in prevalence and incidence of MS during the COVID-19 pandemic occurred not only in PLWH but also in the general population, with a rapid onset of MS even in subjects without previous metabolic changes (15).

In our study, the crude mean changes that were significantly different between the two periods were in lipid and glucose profiles rather than in BMI and weight.

This finding is similar to another study in which the increase in the proportion of individuals with dyslipidemia and DM after the lockdown period was statistically significant and required the addition of new glucose- and lipid-lowering medications (15).

This is probably due to the changes in diet and physical activity that affected our population during the COVID-19 pandemic, which probably had as an immediate consequence a worsening of the lipid and glucose profile rather than a rapid weight gain and change in BMI.

When analyzing the predictive factors for the development of MS in PLWH, we found that age, male sex, BMI, and CD4 cell count were associated with the risk of developing MS. This finding is similar to a previous study that showed how age and a high CD4 cell count were associated with the risk of developing MS (13).

Not only traditional or HIV-related risk factors may lead to the development of MS in PLWH, but antiretroviral therapy has historically been associated with metabolic changes related to fat redistribution and alterations in glucose homeostasis (18).

The current ART regimens are not associated with lipodystrophy and mitochondrial dysfunction, but even the most recent ART therapies have been associated with dyslipidemia, weight gain and insulin resistance (5). In particular, several studies in recent years have described a potential deleterious effect of INSTI-based regimens on insulin resistance and weight gain (5–20). Therefore, we decided to focus on the INSTI-based regimen when analyzing the predictive factors of MS. In our study, there was no difference in the development of MS among subjects exposed to INSTI-containing regimens.

Our study has some limitations that need to be acknowledged. The first is the use of BMI instead of waist circumference in the definition of MS. Unfortunately, waist circumference was not available for the entire cohort. Our decision to use BMI ≥ 30 kg/m^2^ as a surrogate indicator of abdominal obesity may have been associated with an underestimation of MS in the lower BMI ranges, especially considering the problems of body fat distribution observed in PLWH with a long history of therapy and treated with thymidine analogs and/or first-generation protease inhibitors (18).

Second, in contrast to the original NCEP ATP III 2005 definition (4), we included statins, ezetimibe, and fibrates as specific lipid-lowering drugs considered for the definition of MS. This may have resulted in a higher proportion of PLWH diagnosed with MS in our study. However, given the widespread use of these drugs in PLWH with metabolic abnormalities (21), we are confident that the use of these specific treatments may be an appropriate supporting criterion for the diagnosis of MS in our cohort.

Third, although we used the most commonly used definition of MS, it is possible that the use of other diagnostic definitions may have resulted in different prevalence and incidence values of MS in our cohort.

Lastly, the purpose of our study was to compare the prevalence and incidence of metabolic syndrome between the pre-pandemic and pandemic periods. However, our follow-up was interrupted when the lockdown ended but not all social restrictions were lifted. This may have prevented us from seeing further changes in metabolic parameters that might have been observed with longer follow-up.

Despite the aforementioned limitations, one of the strengths of our study is the inclusion of a large number of subjects who were regularly followed, despite the obvious difficulties associated with the pandemic. This makes our results generalizable to similar populations of PLWH in different countries.

## Conclusion

Our study showed a high prevalence of metabolic syndrome in our population and how the pandemic period further worsened the metabolic profile of our cohort, resulting in an increase in both prevalence and incidence of metabolic syndrome.

In conclusion, the COVID-19 pandemic has significantly worsened the risk of MS in PLWH, especially in older men with a good immunologic profile. In this population, early diagnosis and strategies to prevent metabolic syndrome are now urgently needed.

## Data availability statement

The raw data supporting the conclusions of this article will be made available by the authors, without undue reservation.

## Ethics statement

This study involving humans was approved by San Raffaele Scientific Institute Ethics Committee, Milan. This study was conducted in accordance with the local legislation and institutional requirements. The participants provided their written informed consent to participate in this study.

## Author contributions

VS, AC, RP, and LG contributed to the conception and design of the study and data collection. VS, LG, and RP wrote the first version of the manuscript. LG and AP performed data cleaning and statistical analyses. CM, TC, MB, NG, and SN contributed to data collection and revision of the manuscript. All authors contributed to the article and approved the submitted version.

## Funding

This study was partially supported by the 2021 Gilead Fellowship Program.

## Conflict of interest

The authors declare that the research was conducted in the absence of any commercial or financial relationships that could be construed as a potential conflict of interest.

## Publisher’s note

All claims expressed in this article are solely those of the authors and do not necessarily represent those of their affiliated organizations, or those of the publisher, the editors and the reviewers. Any product that may be evaluated in this article, or claim that may be made by its manufacturer, is not guaranteed or endorsed by the publisher.
